# A dataset of hemoglobin blood value and photoplethysmography signal for machine learning-based non-invasive hemoglobin measurement

**DOI:** 10.1016/j.dib.2023.109823

**Published:** 2023-11-18

**Authors:** Tomy Abuzairi, Ester Vinia, Muhammad Arkana Yudhistira, Mia Rizkinia, Winda Eriska

**Affiliations:** a Electrical Engineering, Department of Electrical Engineering, Faculty of Engineering, Universitas Indonesia, Depok 16424, Indonesia; b Biomedical Engineering, Department of Electrical Engineering, Faculty of Engineering, Universitas Indonesia, Depok 16424, Indonesia; c Computer Engineering, Department of Electrical Engineering, Faculty of Engineering, Universitas Indonesia, Depok 16424, Indonesia; d Faculty of Nursing, Universitas Indonesia, Depok 16424, Indonesia

**Keywords:** Hemoglobin, Photoplethysmography, Non-invasive, Machine learning, Dataset

## Abstract

Hemoglobin (Hb), a protein found within red blood cells, is responsible for transporting oxygen and carbon dioxide gasses. A low concentration of Hb indicates the existence of anemia. Traditional invasive Hb examination methods are accurate but have drawbacks, such as pain. A new approach, non-invasive photoplethysmography (PPG), addresses these issues and allows real-time Hb examination. In this article, the dataset consists of PPG signal, gender, age, and Hb value. The PPG signal was measured by a MAX30102 module sensor that emitted two types of light (red and infra-red light) and measured using a photodetector. Total of 68 subjects (56% female and 44% male) within the age of 18–65 years were collected. The total dataset is 816 data from 68 subjects, which each subject provides 12 sets of red and infra-red light signals. The data were collected at Primary Health Center Jatiuwung, Tangerang City, Banten 15,138, Indonesia. Researchers interested in anemia monitoring and those pursuing the development of non-invasive hemoglobin measurement based on machine learning can leverage this dataset.

Specifications Table

**Table:** 

Subject	Biomedical Engineering
Specific subject area	Non-Invasive Hemoglobin Concentration [1,2]
Data format	Raw dataset PPG signal per subject: csv Pre-processed dataset PPG signal per subject: csv Final dataset PPG signal: csv
Type of data	Table
Data collection	The dataset consists of PPG signal, gender, age, and Hb value. The PPG signal was measured by a MAX30102 sensor (Maxim Integrated, California, USA). The Hb value was measured by a Hb meter, Nesco Multicheck 2® (Bioptik Technology Inc, Miaoli, Taiwan).
Data source location	Primary Health Center Jatiuwung, Tangerang City, Banten 15,138, Indonesia.
Data accessibility	Repository name: Mendeley Data Data identification number: 10.17632/xdrwrh9zbk.2 Direct URL to data: https://data.mendeley.com/datasets/xdrwrh9zbk/2

## 1 Value of the Data


 

• Hemoglobin (Hb), a protein found within red blood cells, is responsible for transporting oxygen and carbon dioxide gasses. A low concentration of Hb indicates the existence of anemia. Traditional invasive Hb examination methods are accurate but have drawbacks, such as pain.
• Photoplethysmography (PPG) are frequently employed as an alternative to invasive measuring devices, offering the advantages of rapid, accurate, painless, and real-time measurements. PPG is an optical technique by measuring light reflection through blood.
• This dataset contains PPG signal value, hemoglobin value, age, and gender.
• This dataset is one of the PPG signal value datasets publicly available for non-invasive hemoglobin measurement.
• Researchers interested in anemia monitoring and those pursuing the development of non-invasive hemoglobin measurement devices can leverage this dataset.

## 2 Data Description

This dataset consists of PPG signal, gender, age, and Hb value, in CSV format. The scope of the data is limited to 68 subjects between the ages of 18–65 years. The data were collected at Primary Health Center Jatiuwung, Tangerang City, Banten 15,138, Indonesia. The total dataset is 816 data from 68 subjects. The dataset is available online at the Mendeley repository. The dataset contains one final dataset and two folders, raw PPG signal values and processed PPG signal values from each subject. Table 1 shows representative visualization of the final dataset.

**Table 1:** Representative visualization of the final dataset.

No	Red (a.u)	Infra Red (a.u)	Gender	Age (year)	Hemoglobin (g/dL)
1	115,965.9	105,722.9	Male	21	17.5
2	115,834.4	105,746.7	Male	21	17.5
3	115,741.5	105,786.0	Male	21	17.5
4	115,707.2	105,848.1	Male	21	17.5
5	115,675.6	105,893.5	Male	21	17.5
…	…	…	…	…	…
812	95,220.2	76,709.8	Female	37	11
813	95,100.1	76,664.0	Female	37	11
814	95,031.5	76,624.6	Female	37	11
815	95,100.9	76,680.3	Female	37	11
816	95,191.2	76,704.6	Female	37	11

To understand dataset's variables and their characteristics, dataset variable description is shown in Table 2. The first two variables, Red and Infra-Red, represent the intensity of absorbed light as measured by a PPG sensor. The units of the intensity are arbitrary units (a.u.) and they are of numeric data type in floating-point format. The Gender variable indicates the gender of each respondent and the type is categorical. Age of each respondent in years and is of numeric data type in integer format. Lastly, Hemoglobin is a target variable, which signifies the concentration of hemoglobin in the respondents' blood and is measured in grams per deciliter (g/dL) as a numeric data type in floating-point format.

**Table 2:** Description of variable.

Variable Name	Description	Units	Type	Role
Red	Intensity of the absorbed red light measured using the sensor	arbitrary units (a.u.)	Numeric (float)	Feature
Infra Red	Intensity of the absorbed infra-red light measured using the sensor	arbitrary units (a.u.)	Numeric (float)	Feature
Gender	Gender of each respondent	–	Categorical	Feature
Age	Age of each respondent	year	Numeric (integer)	Feature
Hemoglobin	Hemoglobin concentration measured using a Hb meter	g/dL	Numeric (float)	Target

## 3 Experimental Design, Materials and Methods

### 3.1 PPG device

**Fig 1:**
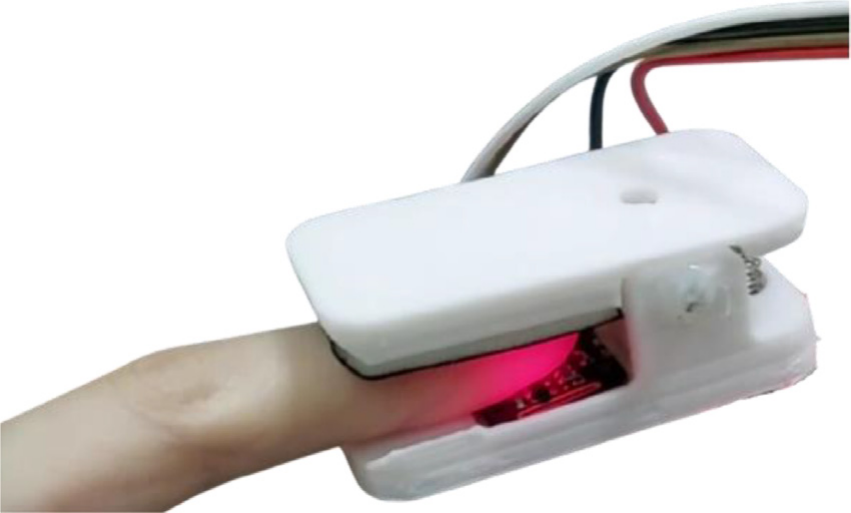
PPG device to measure PPG signal.

PPG signals were recorded using the PPG device depicted in Fig. 1. The PPG device consists of a MAX30102 module sensor (Maxim Integrated, California, USA) and 3-D print case. This PPG case is made in the shape of a fingertip with a total size of 5 cm × 2.5 cm using 3-D Print. The purpose of using this design shape is to reduce bias from external light that could affect the red and infra-red light.

**Fig 2:**
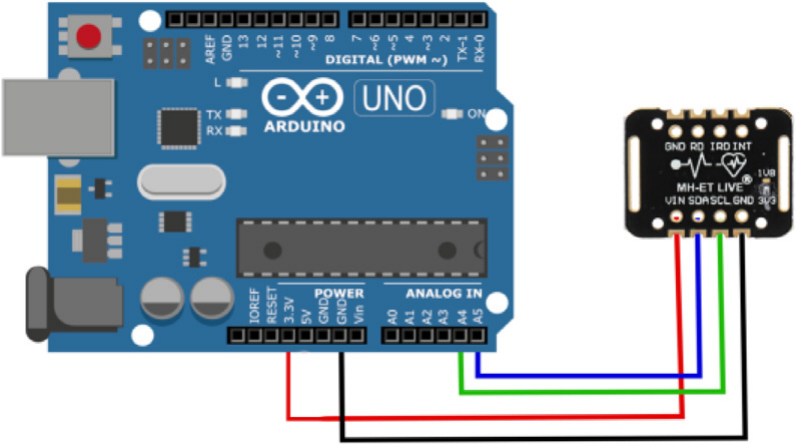
MAX30102 sensor connected to Arduino.

Generally, the MAX30102 sensor measures the oxygen saturation level in the blood and heart rate [3,4]. The MAX30102 sensor incorporates an 18-bit analog-to-digital converter (ADC) and low-noise electronics with ambient light rejection. It operates using two voltage sources, namely 1.8 Volt for the IC and 3.3 Volt for the internal LED. The connection between the MAX30102 sensor and the Arduino Uno (as the microcontroller) is established using the Inter-Integrated Circuit (I2C) protocol, which utilizes two lines: the serial data line (SDA) for data transfer and the serial clock line (SCL) for clock signal transmission. I2C operates on a master-slave connection, where the master device provides commands and reads/writes data while the slave device executes the commands from the master device. The MAX30102 sensor's SDA pin is linked to the A4 pin of the Arduino Uno, while its SCL pin is connected to the A5 pin of the Arduino Uno. Furthermore, the voltage input pin of the MAX30102 sensor is connected to the 3.3 V pin of the Arduino Uno, and its ground pin is connected to the ground pin of the Arduino Uno. Fig. 2 shows the connection between MAX30102 with Arduino Uno.

### 3.2 Dataset collection

The MAX30102 sensor emits two kinds of light: one with a wavelength of 660 nm (red light) and the other with a wavelength of 880 nm (infra-red light). The intensity of both lights absorbed by the finger will be measured using a photodetector. Subsequently, this data is received by the Arduino Uno using the I2C protocol. The 'MAX30105.h' library converts the current values received by the Arduino Uno into infra-red and red light values through the 'getRawValues()' function. This data is then transmitted to a Python program using the 'serial' library through 'serial.Serial()' function, which prompts for the baud rate and port values matching the Arduino Uno. This data collection encompassed 68 subjects. The number of subjects was comparable with other research [1,^5^,6]. The sensor gathered PPG signals at 40 ms intervals for 10 s per subject (250 sets). For the environmental conditions of the data collection, the temperature was at room temperature and the lighting condition was relatively consistent.

The data collection for Hb concentration was conducted using a Hb meter, Nesco Multicheck 2® (Bioptik Technology Inc, Miaoli, Taiwan), and the process was performed invasively by taking a small blood sample. The blood sample was obtained by pricking the finger using a lancet. The blood was then applied to a strip provided on the device. After a few seconds, the device will display the hemoglobin concentration value. This value was recorded and saved in CSV format, along with the previously obtained data on the intensity of infra-red and red light from the PPG device.

A consistent data collection protocol was followed for all subjects, involving several sequential steps: (i) providing a comprehensive explanation of the inform concern in the subjects' native language, (ii) obtaining written consent from the subjects, (iii) gathering demographic information, (iv) conducting invasive-based Hb measurement, and (v) recording non-invasive PPG signals using the sensor.

### 3.3 Pre-processing

**Fig 3:**
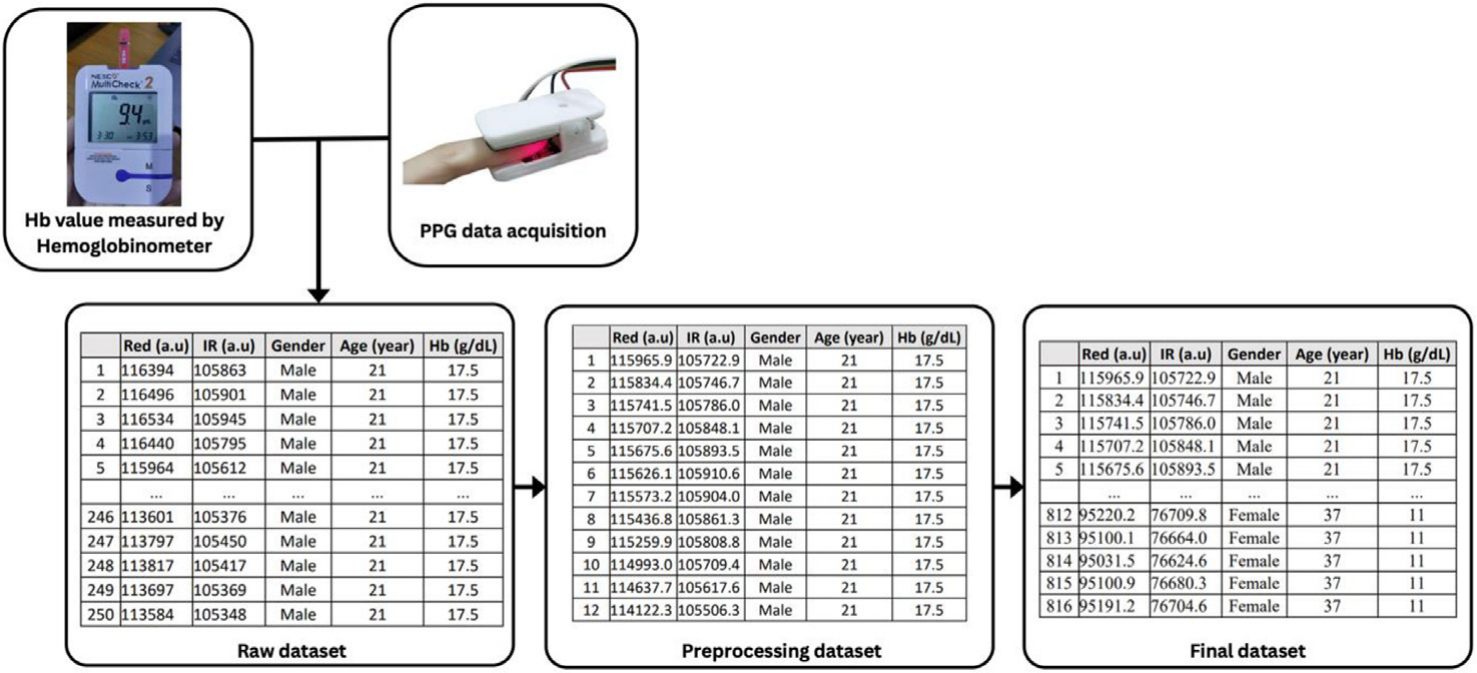
PPG signal data collection.

After the raw PPG signal values were collected at 40 ms intervals over 10 s (250 sets), we averaged them into 12 sets of red and infra-red data. This step was taken to streamline the dataset. To enhance the dataset's utility, specific tasks were executed. The raw PPG signals were analyzed by computing averages of the light data within defined time frames that correspond to the number of peaks in the PPG signal. This procedure yielded uniformly averaged values in each time frame. The total dataset is 816 data from 68 subjects. Subsequently, the consolidated and averaged data was stored as a dataset in a CSV file, ready for subsequent analysis. The PPG signal data collection is illustrated in Fig. 3.

### 3.4 Machine learning

**Fig. 4:**
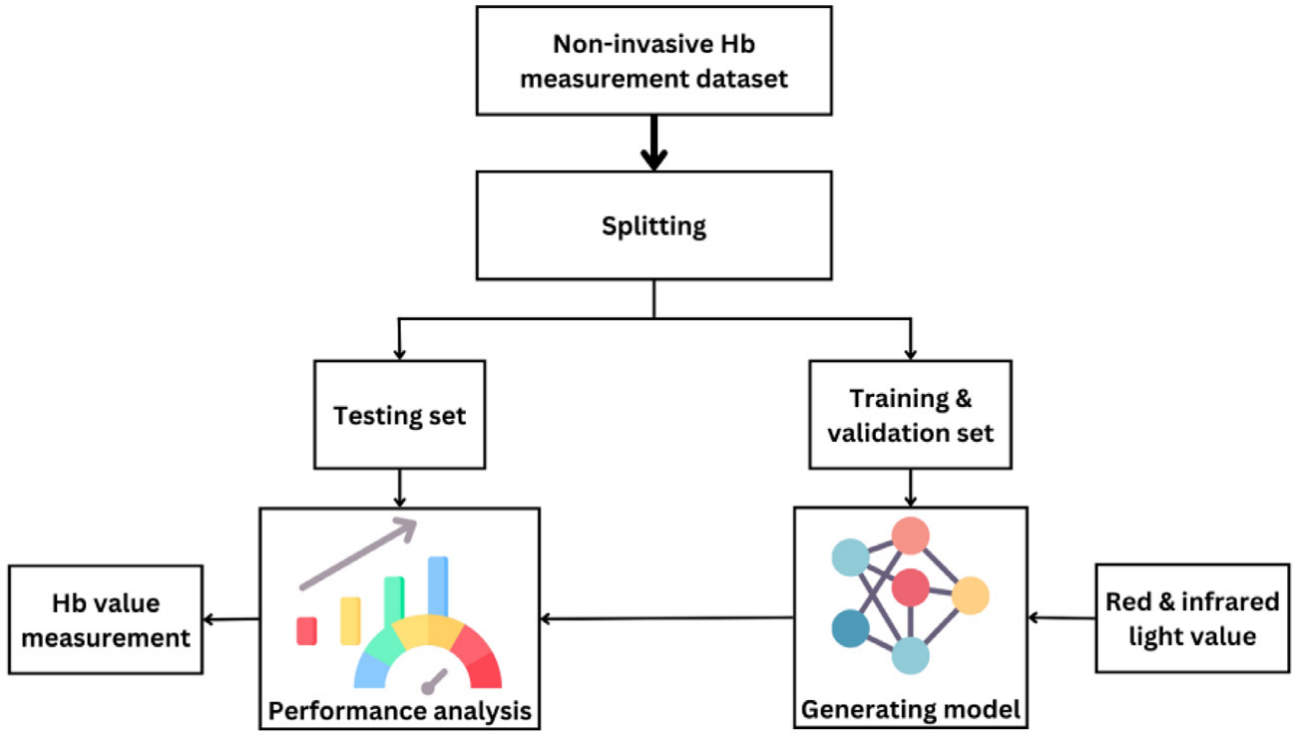
Machine learning model validation of non-invasive Hb measurement.

With technological advancements, several experts have conducted numerous research studies on combining PPG with machine learning for non-invasive Hb measurement [6], [7], [8]. Validating a machine learning model involves assessing the model's performance on a dataset. It involves data pre-processing, data splitting, model training, performance evaluation on a validation set, and model testing on an entirely new test set. This process ensures that the model can generalize to new data and be relied upon for accurate results. The expected model is trained using the training set, and its performance is assessed using the testing set. The steps to validate a machine learning model on the dataset are illustrated in Fig. 4.

## Limitations

The PPG signal has any potential biases from the measurement, such as vibrations and movements, and from the subjects' skin tone. The data has any potential limitations in terms of generalizability to other populations and different demographic groups or age ranges.

## Ethics Statement

Informed consent was obtained from all the individual participants. Data was anonymised, and no personal information, such as phone numbers or email addresses, was requested. They were given the option to withdraw at any point during the study. The Institutional Review Board number for this project is KET-090/UN2.F12.D1.2.1/PPM.00.02/2023 from the Faculty of Nursing Ethics Committee of Universitas Indonesia.

## CRediT authorship contribution statement

**Tomy Abuzairi:** Conceptualization, Methodology, Validation, Writing – original draft, Writing – review & editing, Supervision. **Ester Vinia:** Software, Investigation, Writing – original draft, Visualization. **Muhammad Arkana Yudhistira:** Methodology, Software, Investigation. **Mia Rizkinia:** Validation, Writing – review & editing. **Winda Eriska:** Validation, Writing – review & editing.

## Data Availability

Hemoglobin Photoplethysmography Dataset (Original data) (Mendeley Data)
